# Editorial: Perinatal and post-natal life: metabolism and health outcomes

**DOI:** 10.3389/fendo.2025.1736688

**Published:** 2025-11-17

**Authors:** Ana Paula Santos-Silva, Cintia R. Pinheiro, Natalia da Silva Lima

**Affiliations:** 1 Multidisciplinary Research Center - Xerem in Biological Sciences - NUMPEX-BIO, Federal University of Rio de Janeiro, Duque de Caxias, Rio de Janeiro, Brazil; 2 Integrated Laboratory of Morphofunctional Sciences (LICM), Institute of Sustainability and Biodiversity (NUPEM), Federal University of Rio de Janeiro, Macaé, Rio de Janeiro, Brazil; 3 Regulation of Gene Expression and Applications (EXPRELA) group, Interdisciplinary Centre for Chemistry and Biology – CICA, University of Coruña, A Coruña, Spain

**Keywords:** metabolism, development, DOHaD (development origins of health and disease), pollutans, EDCs

**Figure 1:**
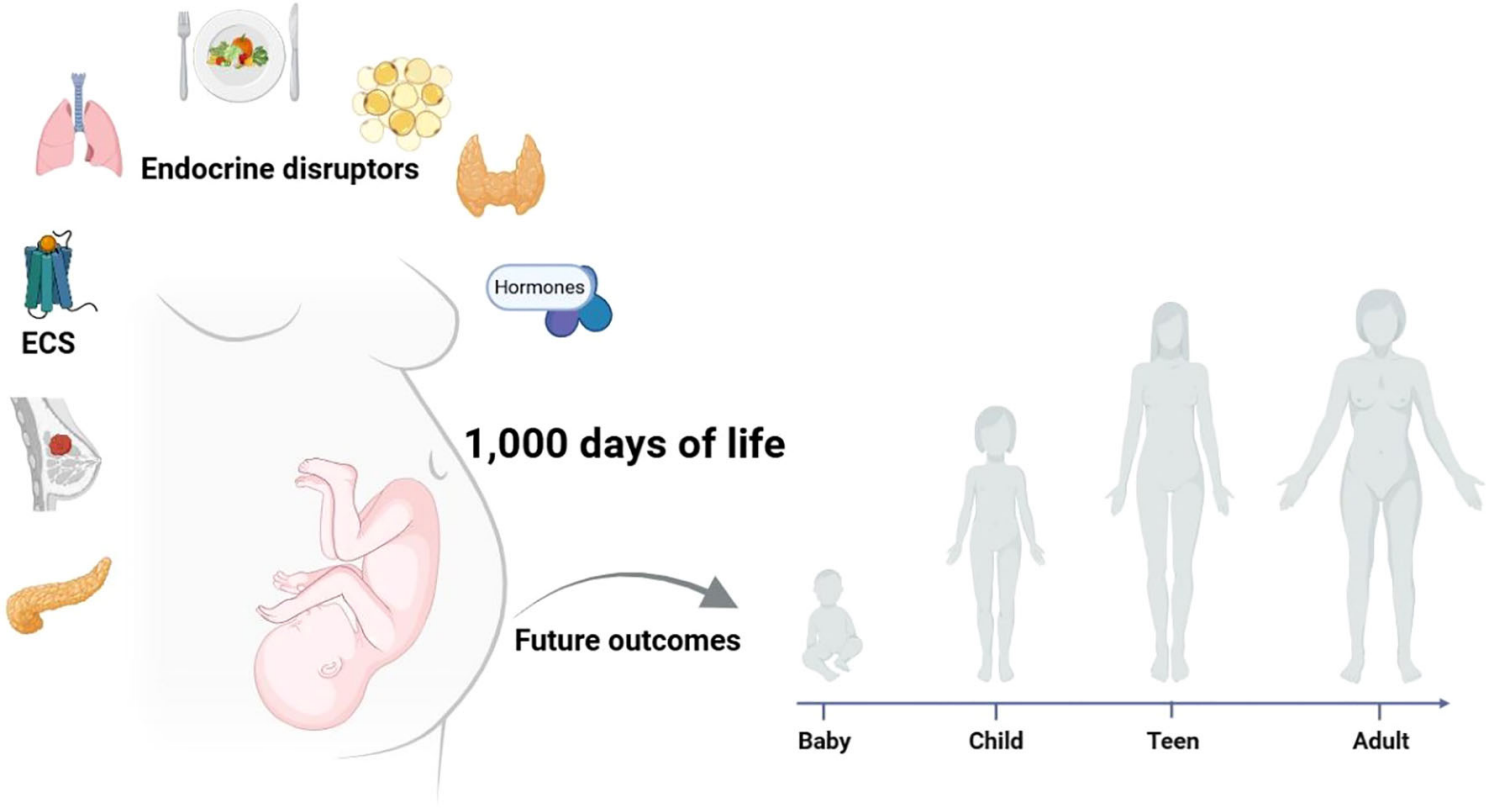
The crucial impact of early developmental stages on lifelong health trajectories underscores the significance of the Developmental Origins of Health and Disease (DOHaD) framework in understanding this topic.

This Research Topic encompasses studies that emphasize the critical role of early life stages in shaping long-term health outcomes, underscoring the relevance of the Developmental Origins of Health and Disease (DOHaD) hypothesis. According to this concept, health and disease trajectories in adulthood are programmed during sensitive developmental windows, particularly within the first 1,000 days of life - from conception through the first two years of age - and during adolescence. Within this framework, exposure to adverse environmental factors, such as dietary restriction or excess, maternal stress, and/or chemical agents, during these early periods of development can influence physiological programming and thereby increase susceptibility to chronic non-communicable diseases (NCDs) across the lifespan. The **Figure 1** illustrates the broad range of environmental, hormonal, and nutritional factors that, when altered during critical stages of development, may disrupt normal programming and predispose individuals to pathological outcomes in adulthood (**Figure 1**).

Within this context, Ning et al. reviewed the relationship between adipose tissue function and reproductive outcomes during adolescence. In this regard, alterations in adipose tissue development, such as those associated with obesity, during this critical period may impair reproductive maturation, leading to disorders ranging from delayed sexual maturation to infertility.

In addition, obesity, insulin intolerance and diabetes are comorbidities that may arise from insults occurring during critical windows of development. Diabetes may represent a long-term outcome of metabolic insults occurring during the early stages of development, but it can also act as an early stress factor, compromising healthy development over the long term. In this context, Lin et al. explored gestational diabetes and its association with an increased risk of adverse outcomes during pregnancy, delivery, and the postpartum period, including preeclampsia, preterm birth, and low birth weight.

Regarding early-life insults and glycemic homeostasis, cases of neonatal hypoglycemia may be associated with infants born to diabetic or obese mothers, as well as with small-for-gestational-age, large-for-gestational-age, or premature infants. In addition to these causes, neonatal hypoglycemia can also result from hyperinsulinemic hypoglycemia, which may have a genetic origin, such as a mutation in the HNF1A (gene encoding hepatocyte nuclear factor α). A case report presented by Chandran et al. described a neonate with an HNF1A gene mutation diagnosed with hyperinsulinemic hypoglycemia. In this case, the mutation was shared by both the neonate and the mother and was associated with hypersensitivity to diazoxide, a pancreatic insulin release inhibitor, requiring dose adjustments for appropriate clinical management. This study highlights that the early stages of development are extremely sensitive to both environmental and genetic insults, which may permanently compromise an individual’s healthy development.

About perinatal insults and environmental pollutants, Juarez et al. investigated the relationship between exposure to brominated flame retardants (BFRs) during gestation and lactation with the development mammary tumors induced by of 7,12-dimethylbenz[a]anthracene (DMBA). Although the number and total volume of tumors were not significantly affected, a marked delay in tumor onset and growth was observed in rats exposed to BFRs, along with alterations in the distribution of molecular subtypes, including an increased incidence of triple-negative tumors. The authors suggest that early-life exposure to endocrine-disrupting compounds such as BFRs may modify the susceptibility window of the mammary gland, thereby influencing its response to carcinogens and the resulting tumor profile. This study underscores the complex interplay between environmental contaminants and breast cancer risk, highlighting the importance of elucidating the long-term effects of these substances on mammary gland programming and carcinogenesis.

Within the broader perspective of how the early hormonal and metabolic environment shapes health across the lifespan, Pelizzo et al. explore the role of endogenous hormones in lung development and function from the fetal period through adulthood. The article reviews evidence indicating that glucocorticoids, thyroid hormones, insulin, ghrelin, leptin, glucagon-like peptide-1 (GLP-1), retinoids, cholecalciferol sex steroids, adipose tissue–derived hormones, and factors such as granulocyte-macrophage colony-stimulating factor (GM-CSF) and glucagon are key regulators of respiratory mechanics and inflammatory processes. Proper maternal hormonal regulation is essential for alveolar maturation, whereas endocrine imbalances may increase susceptibility to respiratory and inflammatory diseases during childhood and later life. The study underscores the importance of integrating endocrinology into the understanding of respiratory physiology, highlighting how sex- and metabolism-related differences shape distinct pulmonary responses. Thus, elucidating early hormonal effects is critical for developing personalized therapeutic strategies in pediatric medicine.

The paper published by Fradet et al. highlights the importance of the endocannabinoid system in the regulation of energy balance, metabolism, and inflammation. According to previous authors, bioactive lipids from this system — such as N-acylethanolamines (NAEs) and 2-monoacylglycerols (2-MAGs) — are found in human breast milk and may influence infant growth and metabolic programming. In the paper, the authors described that infants born to mothers with gestational diabetes mellitus (GDM) are at higher risk of excessive growth and obesity later in life. The study investigated whether GDM alters the profile of endocannabinoid mediators in human milk and how these modifications can change to infant growth at two months postpartum.

GDM appears to modify the biochemical composition of breast milk, influencing the amount of endocannabinoid-related lipids infants receive. Because these compounds regulate appetite, fat metabolism, and energy balance, they could mediate early metabolic programming in infants — potentially affecting obesity risk later in life. These findings emphasize that maternal metabolic health during pregnancy directly shapes the infant’s nutritional and biochemical environment, even after birth.

The other paper, published by Zhang et al. highlights that thyroid diseases - including autoimmune hyperthyroidism, autoimmune hypothyroidism, thyroid nodules (TNs) and thyroid cancer (TC) - are common and have significant health impact. Previous observational studies suggested associations between circulating vitamin levels (A, B_9_, B_12_, C, D, E) and thyroid disease risk, but results have been inconsistent and confounded by reverse causality or other biases. Therefore, the authors aimed to assess causal relationships (and also reverse causality) between circulating vitamin levels and thyroid diseases using a two-sample, bidirectional Mendelian Randomization (MR) design. According to the authors, higher vitamin C levels may protect against autoimmune hypothyroidism, while thyroid disorders can alter vitamin A and D status; these findings highlight possible nutritional pathways in thyroid disease but require clinical trials to confirm causality and therapeutic potential.

